# Knowledge and possession of take-home naloxone kits among street-involved youth in a Canadian setting: a cohort study

**DOI:** 10.1186/s12954-017-0206-6

**Published:** 2017-12-22

**Authors:** Julia Goldman-Hasbun, Kora DeBeck, Jane A. Buxton, Ekaterina Nosova, Evan Wood, Thomas Kerr

**Affiliations:** 1 British Columbia Centre on Substance Use, 608-1081 Burrard Street, Vancouver, V6Z 1Y6 BC Canada; 2 School of Public Policy, Simon Fraser University, 515 West Hastings Street, Vancouver, V6B 5K3 BC Canada; 3 School of Population and Public Health, University of British Columbia, 2206 East Mall, Vancouver, V6T 1Z3 BC Canada; 4 British Columbia Centre for Disease Control, 655 West 12th Avenue, Vancouver, V5Z 4R4 BC Canada; 5 Department of Medicine, University of British Columbia, St. Paul’s Hospital, 608-1081 Burrard Street, Vancouver, V6Z 1Y6 BC Canada

**Keywords:** Street youth, Urban context, Harm reduction, Opioid use, Naloxone

## Abstract

**Background:**

The distribution of take-home naloxone (THN) kits has been an important strategy in reducing overdose fatalities among people who use drugs. However, little is known about the use of THN among youth who are street-involved. The present study explores knowledge and possession of THN among street-involved youth in a Canadian setting.

**Methods:**

Data were derived from the At-Risk Youth Study (ARYS), a prospective cohort of street-involved youth age 14–28 at enrollment in Vancouver, Canada. Participants completed a standardized questionnaire, which included items related to knowledge and possession of THN, sociodemographic characteristics, and substance use-related factors. Multivariable logistic regression models were used to identify factors independently associated with knowledge and possession of THN.

**Results:**

Between December 2014 and November 2016, 177 youth were interviewed, including 68 females (38.4%). While 126 (71.2%) participants reported knowledge of THN, only 40 (22.6%) possessed a THN kit. Caucasian/white ethnicity was found to be positively associated with both knowledge and possession of THN (both *p* < 0.05). Public injection drug use in the last 6 months was found to be positively associated with knowledge of THN, while daily heroin use and daily methamphetamine use were associated with possession of THN (all *p* < 0.05). Male gender was negatively associated with possession of THN (*p* < 0.05).

**Conclusions:**

These findings highlight important gaps between knowledge and possession of THN among youth and the need to increase participation in THN programs among specific populations including non-white and male youth. Further research is needed to gain a better understanding of the barriers that may prevent certain youth from acquiring THN kits.

## Background

North America is in the midst of an escalating overdose epidemic. Increases in overdose deaths have been driven by both prescription and non-prescription opioids in the past two decades, with a 200% increase in the rate of opioid-related overdose deaths reported in the United States (U.S.) since the year 2000 [1]. In British Columbia (B.C.), illicit drug overdose deaths increased by 49% from 2016 to September 2017, and many of these deaths involved opioids [2]. In addition, the rate of overdose deaths involving synthetic opioids other than methadone, such as fentanyl, increased by 80% from 2013 to 2014 in the U.S. [1]. Fentanyl was detected in 68% of illicit drug overdose deaths in 2016 in B.C., and these rates are increasing: preliminary data suggests that the proportion of illicit drug overdose deaths for which fentanyl was detected was 83% from January to September 2017 [2]. Opioid use is also a concern among youth: while there is little national Canadian data on opioid use among youth, one study using survey data from a nationally representative sample of youth aged 10–18 in the U.S. found the 30-day prevalence of opioid use to be 4.8% from 2008 to 2011 [3].

Take-home naloxone (THN) programs, such as B.C.’s Take Home Naloxone program [4] and the Overdose Education and Nasal Naloxone Distribution program (OEND) in Massachusetts [5], have been important strategies in reducing overdose deaths among persons who use drugs. Naloxone is an opioid antagonist used to temporarily reverse the effects of an overdose [6]. Naloxone distribution programs provide participants with naloxone in a packaged kit and also provide training on how to recognize and respond to an opioid-related overdose, including administration of naloxone [6]. In 2016, THN programs were operational in seven of Canada’s 13 provinces and territories [6]. Similar programs have also been implemented in Asia, Australia, Europe, and the U.S. [7]. A recent systematic review suggested that THN programs are effective in reducing overdose mortality rates in program participants and in the community [8], with little risk involved in its administration [9]. However, there is evidence that younger people who use drugs (PWUD) are less likely to access harm reduction services than older PWUD [10–12], which raises concerns about the uptake of THN among youth.

Previous studies have evaluated THN program implementation in various settings [13–15]. One study examining the perceptions of homeless and precariously housed youth regarding the THN program in Vancouver, B.C., found that participants reported positive experiences with THN programs—such as increased sense of safety and improved self-esteem—and expressed the importance of increasing access to THN programs [16]. In addition, in a 1999 study assessing the possible impact and acceptability of a THN program in South London, 89% of participants who had witnessed an overdose fatality reported that they would have administered naloxone if it had been available [17]. However, to our knowledge, there have been no quantitative reports of participation rates in THN programs, or of the factors associated with participation, among youth who are street-involved.

Drawing on a prospective cohort study of street-involved youth in a Canadian setting, we undertook the present study to identify sociodemographic and substance use-related factors associated with knowledge and possession of THN among street-involved youth in Vancouver, B.C. Vancouver is home to one of Canada’s largest and longest operational provincial THN programs: the program was launched in 2012 and has since distributed over 54,000 kits, with a significant increase in distribution since 2016 (from 3394 kits in 2015 to 22,494 kits in 2016) [4]. B.C.’s THN program targets people who are at risk of an opioid overdose and people who are likely to witness and respond to an overdose (e.g., family or friend of someone at risk) [4].

## Methods

The At-Risk Youth Study (ARYS) is an open prospective cohort study of street-involved youth who use illicit drugs based in Vancouver, Canada. Youth aged 14–28 who have used any illicit drug (other than or in addition to cannabis) in the preceding 30 days are eligible for study enrollment. Recruited youth are street-involved at baseline, defined as having been without stable housing or having accessed street-based services in the preceding 6 months [18–20]. Street-based outreach is used to enhance study recruitment both during daytime and nighttime hours in a range of neighborhoods throughout Vancouver where street youth are known to congregate. Snowball sampling is also used to maximize study enrollment. After providing informed consent, participants complete an interviewer-administered questionnaire regarding sociodemographic and socioeconomic details, engagement with health and social services, substance use patterns, and other behavioral data. All participants are provided with monetary compensation for their time ($30 CAN). ARYS is approved by the University of British Columbia and Providence Health Care Research Ethics Board. The study has been described in more detail in previous publications [21].

Questions regarding THN were added to the cohort questionnaire in December 2014. The present analysis draws on baseline data from all ARYS participants who completed a study visit between December 2014 and November 2016. Knowledge and possession of THN were the main outcomes of interest and were ascertained through the following questions: “Have you heard about a take-home Narcan rescue kit that you can keep with you for an opiate overdose?” and “Do you currently own a take-home Narcan rescue kit?” Individuals who responded “yes” versus “no” to these questions were compared using logistic regression as described below.

Explanatory variables of interest were chosen a priori based on what we hypothesized might increase awareness and possession of THN. We examined the following explanatory variables: age (continuous, per year older), gender (male vs. female); residing in Vancouver’s Downtown Eastside (DTES) neighborhood in the last 6 months [a district with an open drug market as well as high levels of substance use, poverty, and homelessness] (yes vs. no); ever absolutely homeless (yes vs. no); education status (high school or greater vs. other); employment in the last 6 months (yes vs. no); daily heroin use in the last 6 months (yes vs. no); daily methamphetamine use in the last 6 months (yes vs. no); daily prescription opioid use in the last 6 months (yes vs. no); daily cocaine or crack use in the last 6 months (yes vs. no); public injection drug use in the last 6 months (yes vs. no); ever non-fatal overdosed [negative reaction from using too much drugs] (yes vs. no); incarceration in the last 6 months, defined as being in detention, prison, or jail overnight or longer (yes vs. no); unable to access addiction treatment, defined as responding affirmatively to the question: “In the past 6 months, have you tried to access any treatment program but were unable?” (yes vs. no); currently in methadone/methadose treatment (yes vs. no); and been in alcohol or drug treatment in last 6 months (yes vs. no).

Initially, we examined the descriptive characteristics, stratified by our two outcomes of interest (i.e., knowledge and possession of THN) at the first study visit. Next, we examined the bivariable associations between each explanatory variable and our two outcomes of interest using logistic regression. As a last step, we fitted multivariable models, considering all variables in bivariable analyses as the full model. All statistical analyses were performed using R, version 3.2.4 (R Foundation for Statistical Computing, Vienna, Austria). All *p* values were two-sided, and tests were considered significant at *p* < 0.05 level.

## Results

Between December 2014 and November 2016, a total of 177 participants completed a baseline survey: 3 (1.7%) completed a survey in 2014, 60 (33.9%) completed a survey in 2015, and 114 (64.4%) completed a survey in 2016. Among this sample, 68 (38.4%) identified as female, 97 (54.8%) identified as Caucasian/white, and the median age was 22.1 years (inter-quartile range [IQR] = 20.2–23.4 years). Overdose was common in our study sample, with 81 (45.8%) participants having ever experienced a non-fatal overdose.

Table 1 reports baseline characteristics of all participants stratified by our two main outcomes of interest. As shown, 126 (71.2%) study participants reported knowledge of THN, while 40 (22.6%) reported possession of a THN kit. In addition, when stratified by year, 34 participants (54%) reported knowledge of THN in 2014 and 2015 (combined), while 92 participants (80.7%) reported knowledge of THN in 2016. Six participants (9.5%) reported possession of THN in 2014 and 2015 (combined), while 34 participants (29.8%) reported ownership of THN in 2016.

**Table 1:** Baseline characteristics of 177 street-involved youth, stratified by knowledge and possession of THN

	Knowledge of THN		Possession of THN	
Characteristic	Yes (%) *n* = 126	No (%) *n* = 51	Yes (%) *n* = 40	No (%) *n* = 137
Sociodemographic characteristics				
Median age (Q1–Q3)^a^	22.2 (20.2–23.4)	21.9 (19.6–23.4)	22.1 (20.1–23.7)	22.1 (20.2–23.4)
Male gender	73 (57.9)	36 (70.6)	15 (37.5)	94 (68.6)
White ethnicity	78 (61.9)	19 (37.3)	29 (72.5)	68 (49.6)
Downtown Eastside residence^b^	33 (26.2)	14 (27.5)	8 (20.0)	39 (28.5)
Ever absolutely homeless	116 (92.1)	41 (80.4)	36 (90.0)	121 (88.3)
High school completion	58 (46.0)	26 (51.0)	20 (50.0)	64 (46.7)
Employment^b^	29 (23.0)	14 (27.5)	10 (25.0)	33 (24.1)
Incarceration^b^	22 (17.5)	4 (7.8)	6 (15.0)	20 (14.6)
Substance use-related factors				
Daily heroin use^b^	44 (34.9)	6 (11.8)	20 (50.0)	30 (21.9)
Daily cocaine or crack use^b^	4 (3.2)	6 (11.8)	1 (2.5)	9 (6.6)
Daily methamphetamine use^b^	37 (29.4)	12 (23.5)	17 (42.5)	32 (23.4)
Daily use of prescription opioids^b^	10 (7.9)	2 (3.9)	1 (2.5)	11 (8.0)
Public injecting^b^	61 (48.4)	7 (13.7)	22 (55.0)	46 (33.6)
Ever overdosed	61 (48.4)	20 (39.2)	20 (50.0)	61 (44.5)
Drug or alcohol treatment^b^	77 (61.1)	22 (43.1)	27 (67.5)	72 (52.6)
Unable to access addiction treatment^b^	28 (22.2)	4 (7.8)	7 (17.5)	25 (18.2)

^a^Q1–Q3 = 25th–75th percentiles

^b^In the last 6 months

Table 2 shows the results of the bivariable and multivariable analyses for knowledge of THN. In bivariable analyses, factors positively associated with knowledge of THN included Caucasian/white ethnicity, ever homeless, daily heroin use, public injection drug use, recent engagement in drug or alcohol treatment, and inability to access addiction treatment. Daily cocaine or crack use was negatively associated with knowledge of THN. In multivariable analyses, Caucasian/white ethnicity (adjusted odds ratio [AOR] = 2.36, 95% confidence interval [CI] 1.02–5.61) and public injection drug use (AOR = 5.61, 95% CI 1.90–19.12) remained independently positively associated with knowledge of THN. Daily cocaine or crack use (AOR = 0.09, 95% CI 0.01–0.59) remained independently negatively associated with knowledge of THN.

**Table 2:** Bivariate and multivariate logistic regression analyses of factors associated with knowledge of THN

Characteristics	Unadjusted		Adjusted	
	Odds ratio (95% CI)	*p* value	Odds ratio (95% CI)	*p* value
Age (per year younger)	1.06 (0.94–1.20)	0.352	1.05 (0.88–1.26)	0.587
Gender (male vs. female)	0.57 (0.28–1.14)	0.119	0.61 (0.24–1.49)	0.283
White ethnicity (yes vs. no)	2.74 (1.41–5.43)	0.003	2.36 (1.02–5.61)	0.047
Downtown Eastside residence^a^ (yes vs. no)	0.94 (0.46–1.99)	0.863	0.43 (0.15–1.19)	0.108
Ever homeless (yes vs. no)	2.83 (1.09–7.38)	0.031	1.99 (0.54–7.34)	0.297
High school completion (yes vs. no)	0.82 (0.43–1.57)	0.551	0.94 (0.40–2.20)	0.883
Employment^a^ (yes vs. no)	0.79 (0.38–1.69)	0.534	0.91 (0.36–2.37)	0.842
Incarceration^a^ (yes vs. no)	2.43 (0.87–8.67)	0.120	1.84 (0.50–8.08)	0.380
Daily heroin use^a^ (yes vs. no)	4.02 (1.70–11.17)	0.003	2.37 (0.73–9.03)	1.171
Daily cocaine or crack use^a^ (yes vs. no)	0.25 (0.06–0.90)	0.036	0.09 (0.01–0.59)	0.016
Daily methamphetamine use^a^ (yes vs. no)	1.35 (0.65–2.95)	0.433	0.70 (0.26–1.90)	0.477
Daily use of prescription opioids^a^ (yes vs. no)	2.11 (0.53–14.07)	0.346	1.38 (0.17–18.75)	0.784
Public injecting^a^ (yes vs. no)	6.09 (2.69–15.72)	< 0.001	5.61 (1.90–19.12)	0.003
Ever overdosed (yes vs. no)	1.41 (0.73–2.77)	0.314	0.76 (0.32–1.76)	0.522
Unable to access addiction treatment^a^ (yes vs. no)	3.29 (1.20–11.58)	0.035	3.41 (1.02-14.10)	0.062
Drug or alcohol treatment^a^ (yes vs. no)	2.00 (1.03–3.91)	0.041	1.71 (0.71–4.19)	0.235

*CI* confidence interval

^a^In the last 6 months

Table 3 shows the results of the bivariable and multivariable analyses for possession of THN. In bivariable analyses, factors positively associated with possession of THN included: Caucasian/white ethnicity; daily heroin use; daily methamphetamine use; and public injection drug use. Male gender was negatively associated with possession of THN. In multivariable analyses, Caucasian/white ethnicity (AOR = 2.51, 95% CI 1.02–6.51), daily heroin use (AOR = 3.08, 95% CI 1.09–9.11), and daily methamphetamine use (AOR = 2.99, 95% CI 1.13–8.13) remained independently positively associated with possession of THN. Male gender (AOR = 0.29, 95% CI 0.11–0.72) remained independently negatively associated with possession of THN.

**Table 3:** Bivariate and multivariate logistic regression analyses of factors associated with ownership of THN

Characteristics	Unadjusted		Adjusted	
	Odds ratio (95% CI)	*p* value	Odds ratio (95% CI)	*p* value
Age (per year younger)	1.04 (0.90–1.20)	0.610	1.05 (0.88–1.25)	0.627
Gender (male vs. female)	0.27 (0.13–0.57)	0.001	0.29 (0.11–0.72)	0.008
White ethnicity (yes vs. no)	2.68 (1.27–5.99)	0.012	2.51 (1.02–6.51)	0.049
Downtown Eastside residence^a^ (yes vs. no)	0.63 (0.25–1.43)	0.289	0.38 (0.11–1.14)	0.097
Ever homeless (yes vs. no)	1.19 (0.41–4.35)	0.768	1.25 (0.31–6.00)	0.767
High school completion (yes vs. no)	1.14 (0.56–2.32)	0.714	1.37 (0.54–3.54)	0.508
Employment^a^ (yes vs. no)	1.05 (0.45–2.32)	0.906	1.23 (0.44–3.31)	0.685
Incarceration^a^ (yes vs. no)	1.02 (0.35–2.62)	0.963	0.80 (1.19–2.96)	0.748
Daily heroin use^a^ (yes vs. no)	3.57 (1.70–7.54)	0.001	3.08 (1.09–9.11)	0.036
Daily cocaine or crack use^a^ (yes vs. no)	0.36 (0.02–2.03)	0.346	0.25 (0.01–2.43)	0.295
Daily methamphetamine use^a^ (yes vs. no)	2.43 (1.15–5.09)	0.019	2.99 (1.13–8.13)	0.028
Daily use of prescription opioids^a^ (yes vs. no)	0.29 (0.02–1.58)	0.248	0.20 (0.01–1.36)	0.161
Public injecting^a^ (yes vs. no)	2.53 (1.23–5.29)	0.012	1.55 (0.55–4.32)	0.403
Ever overdosed (yes vs. no)	1.23 (0.61–2.50)	0.566	0.63 (0.24–1.58)	0.336
Unable to access addiction treatment^a^ (yes vs. no)	0.94 (0.35–2.28)	0.899	0.77 (0.22–2.47)	0.671
Drug or alcohol treatment^a^ (yes vs. no)	1.85 (0.89–3.98)	0.106	1.47 (0.58–3.82)	0.419

*CI* confidence interval

^a^In the last 6 months

## Discussion

In the present study of street-involved youth, we observed an important gap between reported knowledge of THN programs and reported possession of THN kits. We also found an increase in the rates of both knowledge and possession of THN that parallels the increase in THN distribution that occurred in 2016. Caucasian/white ethnicity was the only variable found to be independently positively associated with both knowledge and ownership of THN. Public injection drug use was independently positively associated with THN knowledge, while daily cocaine or crack use was independently negatively associated with THN knowledge. In addition, daily heroin and methamphetamine use were independently positively associated with THN ownership, while male gender was independently negatively associated with THN ownership.

Our findings build on a qualitative study conducted in Vancouver, B.C., which found THN programs to be generally well-received among street-involved youth, though the study did not assess rates of uptake in this population [16]. Our findings also build on previous studies that have examined knowledge of and participation in THN programs among PWUD [8, 22]. One recent study examining THN participation among adult PWUD in Vancouver, B.C., also identified an alarming gap between the rates of knowledge and possession of THN [22]. One likely explanation for this gap is an underestimation of overdose risk (both personal and witnessed) among PWUD. There is in fact evidence that PWUD are likely to underestimate their own risk of opioid overdose [23], which has been identified as a major barrier to THN ownership among adult PWUD [22]. Interestingly, one study in the U.S. found that people who use opioids reported their desire to help an overdosing peer to be a bigger motivation for THN enrollment than a fear of personal overdose [24], further suggesting that PWUD tend to underestimate their risk of personal overdose and may also be underestimating their risk of witnessing an overdose. It is also possible that some youth did not participate in the THN program due to perceived ineligibility (at the time of data collection only individuals considered high-risk for witnessing or experiencing an overdose received free kit following administration training), though this is unlikely due to the high-risk study population, as well as the high rates of non-fatal overdose among this sample of youth. Nolan et al.’s [22] study of adult PWUD identified even lower rates of THN possession (13%) than those reported in our sample of young PWUD (22.5%). This may partially be explained by the fact that naloxone distribution increased significantly in 2016; Nolan et al.’s [22] study used data from 2014 to 2015 only, while the current study included data from participants who completed a questionnaire in 2016. However, it is also worth noting that Vancouver’s THN program targeted youth through the distribution of THN in emergency departments as well as youth-centered promotional activities [25], which may have contributed to higher rates of uptake among youth.

Public injection, heroin use, and methamphetamine use have all been associated with non-fatal overdose [26, 27] and were also associated with THN knowledge or possession in the present study. This may suggest that certain forms of high-intensity drug use can prompt THN participation. The present study also found that cocaine or crack use was negatively associated with THN knowledge, which could be explained by a lower perceived risk of opioid-related overdose among people who use crack or cocaine when compared to people who use opioids. Because naloxone only reverses the effects of opioid-related overdoses (and not overdoses caused by cocaine or crack), naloxone kit acquisition may seem irrelevant to youth who do not use opioids daily. However, all street-involved youth are at risk of witnessing an overdose due to the high rates of daily opioid use and non-fatal overdose in their environment [28], and one study of youth who inject drugs found that “speedball” (a mixture of heroin and cocaine) use was associated with an increased risk of overdose [29]. In addition, fentanyl-detected deaths in B.C. often involve the use of other illicit drugs [2], though it remains unclear what proportion of these deaths can be attributed to fentanyl contamination and polysubstance use. As such, all street-involved youth who use illicit drugs would be important THN owners, and youth who use cocaine or crack may benefit from targeted educational and marketing efforts to increase THN participation.

A recent meta-analysis found male gender to be one of the factors most strongly associated with drug overdose death [30], and in B.C., males accounted for 83% of all illicit drug overdose deaths from January to September 2017 [2]. However, male gender was negatively associated with THN ownership in the current study. This highlights the need to target male youth in THN programs, who may face sociocultural barriers to accessing THN programs not experienced by male adult PWUD. There is in fact evidence that male adolescents and young adults are less likely than their female counterparts to access health services due to stigma, as well as gender, social, and cultural norms [31, 32]. The same meta-analysis also found Caucasian/white ethnicity to be positively associated with drug overdose death [30]. In the present study, Caucasian/white ethnicity was positively associated with both knowledge and possession of THN, which is consistent with what has been found in the literature of adult PWUD [22]. However, in B.C., First Nations individuals are at increased risk of fatal overdose compared to the general population [33]. First Nations individuals, and other non-white individuals who may be more vulnerable to overdose, may then also benefit from targeted strategies to improve their access to THN.

There are limitations to this study. First, because this is a cross-sectional study, we are unable to infer causation. Second, because there are no registries of street youth to draw upon, our sample was not randomly selected and therefore may not be representative of all street youth in Vancouver. However, we note that the characteristics of the ARYS sample are similar to those from other studies of high-risk youth [34–36]. Third, we relied on self-report, which may have been subject to response biases, including recall bias and socially desirable responding, though we know of no reason why this would explain the associations we identified in this study. Fourth, due to our smaller sample size, we were not able to include data related to witnessed overdose and naloxone administration, which may have influenced the results of our multivariate analyses. We also did not ask youth about their reasons for not owning kits, or whether those who reported THN possession carried the kits with them. Lastly, our study included pre-2016 data, after which THN distribution increased significantly. Our results demonstrate an increase in THN uptake that parallels the program’s expansion; however, current rates of THN uptake may be continuing to rise.

## Conclusions

To our knowledge, this is the first study to examine sociodemographic characteristics and substance use-related factors of street-involved youth who are familiar with and have acquired THN kits and the first to evaluate the rates of knowledge and possession of THN in this population. The findings of the present study highlight the need to increase knowledge of and access to THN among all street-involved youth who use illicit drugs, particularly among local populations found to be among the highest risk of fatal overdose. Specifically, these findings suggest that males, people who do not identify as “Caucasian/white,” and people who use crack or cocaine would benefit from targeted approaches to improve their access to THN programs. Future research should focus on examining interventions that aim to address barriers to THN acquisition among those currently underrepresented in THN programs.

## Abbreviations

AOR: Adjusted odds ratio
ARYS: At-Risk Youth Study
B.C.: British Columbia
CI: Confidence interval
DTES: Downtown Eastside
OEND: Overdose Education and Nasal Naloxone Distribution program
OR: Odds ratio
PWUD: People who use drugs
THN: Take-home naloxone
U.S.: United States
